# Cost effectiveness, nitrogen, and phosphorus removal in field-based woodchip bioreactors treating agricultural drainage water

**DOI:** 10.1007/s10661-023-11358-8

**Published:** 2023-06-16

**Authors:** Finn Plauborg, Maja H. Skjødt, Joachim Audet, Carl C. Hoffmann, Brian H. Jacobsen

**Affiliations:** 1 Department of Agroecology, Aarhus University, Blichers Allé 20, 8830 Tjele, DK Denmark; 2 WATEC, Aarhus University Centre for Water Technology, 8000 Aarhus C, Denmark; 3 Department of Ecoscience, C.F. Møllers Allé, Aarhus University, 8000 Aarhus C, Denmark; 4 Department of Food and Resource Economics, University of Copenhagen, Rolighedsvej 23, 1958 Frederiksberg, Denmark

**Keywords:** Environmental measures, Constructed wetlands, N removal, Residence time, Investment, Unit costs

## Abstract

Nitrogen (N) and phosphorus (P) losses to surface and coastal waters are still critically high across Europe and globally. Measures to mitigate and reduce these losses are being implemented both at the cultivated land surface and at the edge-of-fields. Woodchip bioreactors represent a new alternative in Denmark for treating agricultural drainage water, and the present study—based on two years of data from five Danish field-based bioreactors—determined N removal rates varying from 1.49 to 5.37 g N m^−3^ d^−1^ and a mean across all bioreactors and years of 2.90 g N m^−3^ d^−1^. The loss of phosphorus was relatively high the first year after bioreactor establishment with rates varying from 298.4 to 890.8 mg P m^−3^ d^−1^, but in the second year, the rates ranged from 12.2 to 77.2 mg P m^−3^ d^−1^. The investments and the costs of the bioreactors were larger than expected based on Danish standard investments. The cost efficiency analysis found the key issues to be the need for larger investments in the bioreactor itself combined with higher advisory costs. For the four woodchip bioreactors considered in the cost efficiency analysis, the N removal cost was around DKK 350 per kg N ($50 per kg N), which is ca. 50% higher than the standard costs defined by the Danish authorities. Based on the estimated costs of the four bioreactor facilities included in this analysis, a bioreactor is one of the most expensive nitrogen reduction measures compared to other mitigation tools.

**Supplementary Information:**

The online version contains supplementary material available at 10.1007/s10661-023-11358-8.

## Introduction

Agricultural production is a major source of diffuse pollution, which in Europe mostly is due to excessive emissions of nutrients (nitrogen (N) and phosphorus (P)) and chemicals such as pesticides (EEA, 2018). Nutrient enrichment causes eutrophication, which in turn leads to loss of aquatic biodiversity and reduction of fish stocks. Excessive nutrient enrichment can also endanger human health due to, for instance, toxic algal blooms, and can impair the use of water for drinking and bathing (EEA, 2018). Within the European Union (EU), member states have and are currently implementing different kinds of measures to reduce diffuse nutrient pollution to comply with the EU Water Frame Directive (WFD) (WFD, 2000). The measures at farm level include, amongst others, nutrient planning, fertiliser standards (e.g. timing), appropriate tillage and catch crops, buffer strips, and crop rotation. Other tools focus on reducing the N loss from agricultural drainage water such as constructed wetlands that capture and retain nutrient losses (Carstensen et al., 2020; EEA, 2018). In Denmark, more than 50% of the country’s cultivated area is systematically tile drained (Møller et al., 2018; Olesen, 2009). The tile drainage systems act as a fast transport route of dissolved chemicals, for instance nitrate (NO_3_^-^), to recipient surface water bodies by bypassing the soil domain during the wet season or after heavy rainfall events (Motarjemi et al., 2021). Diffusive losses of P with drainage water from agricultural areas to streams and lakes have gained increased attention in Denmark as lakes are generally sensitive to P (Andersen and Heckrath, 2020). According to Dalgaard et al. (2014), in Denmark N losses to the aquatic and atmospheric environment have been significantly reduced; e.g. the flow weighted concentration of total N (TN) showed a 46% decline from an average of 7.1 mg N L^−1^ in 1990–1994 to 3.9 mg N L^−1^ in 2012, but complying with the WFD and Habitats Directives remains a major challenge that calls for new approaches, measures, and technologies to mitigate agricultural N losses and control N flows (Hoffmann et al., 2020). The establishment of free water surface-constructed wetlands and woodchip bioreactors was a central part of the so-called collective measures to be implemented from 2015 to 2021, and the plan was that these would reduce N losses to the sea by 900 t N per year or 15% of the total expected reductions (MST, 2015). However, an assessment made in 2020 showed that the expected effect at the end of 2021 would be only 332 t N per year due to slower implementation than expected (Ministry of Environment & Food, 2020). In the draft version of the coming River Basin Management Plans, it is expected that constructed wetlands will generate a further reduction to the sea of 555 t N per year by 2027 (Ministry of Environment, 2021).

In a woodchip bioreactor, drainage water is routed horizontally or vertically through a basin filled with woodchips before it reaches an outlet (e.g. Hoffmann et al., 2019). It is well established that woodchip bioreactors are efficient at removing N by denitrification and that the efficiency increases with increasing water temperature and hydraulic residence time (HRT) (Addy et al., 2016; Audet et al., 2021; Christianson et al., 2012; Hoffmann et al., 2019; Schipper et al., 2010). As diffusive losses from agricultural areas of P in addition to N have detrimental effects on the aquatic environment, the potential of P removal by woodchip bioreactors has gained increasing attention. For two 100 m^3^ woodchip bioreactors, Carstensen et al. (2019) found that 67–85% of the annual loading of particulate P was retained, but in some years the bioreactors acted as a sink and in other years as a source of phosphate. Similarly, Gosch et al. (2020) found that a bioreactor (76.5 m^3^) turned from acting as a phosphate source in the first year of operation to a phosphate sink in the second and third year.

For woodchip bioreactors to gain wide acceptance as a relevant mitigation measure, it is important to determine their cost-effectiveness as this will have to be evaluated against that of other mitigation measures used in Denmark and around the world (Eriksen et al., 2020; Hoffmann et al., 2020). In particular, it is relevant to analyse to what extent the reduction costs of bioreactors per kg N and P compare with the estimated standard costs used by the Ministry of Environment in Denmark.

Although the efficiency of N removal by woodchip bioreactors is relatively well established, Addy et al. (2016) concluded in a meta-analysis that more field-based studies of the performance of woodchip bioreactors are needed to determine their removal rates in different landscapes at different nitrate loadings and under different climate conditions. Hence, the main objectives of the present study were to (i) present results on N and P removal of five field-based woodchip bioreactor facilities that receive agricultural drainage water and are located in different geo-regions in Denmark and (ii) to present the results of a cost-effectiveness analysis for each reactor facility.

## Materials and methods

### Study sites and weather conditions

**Fig. 1:**
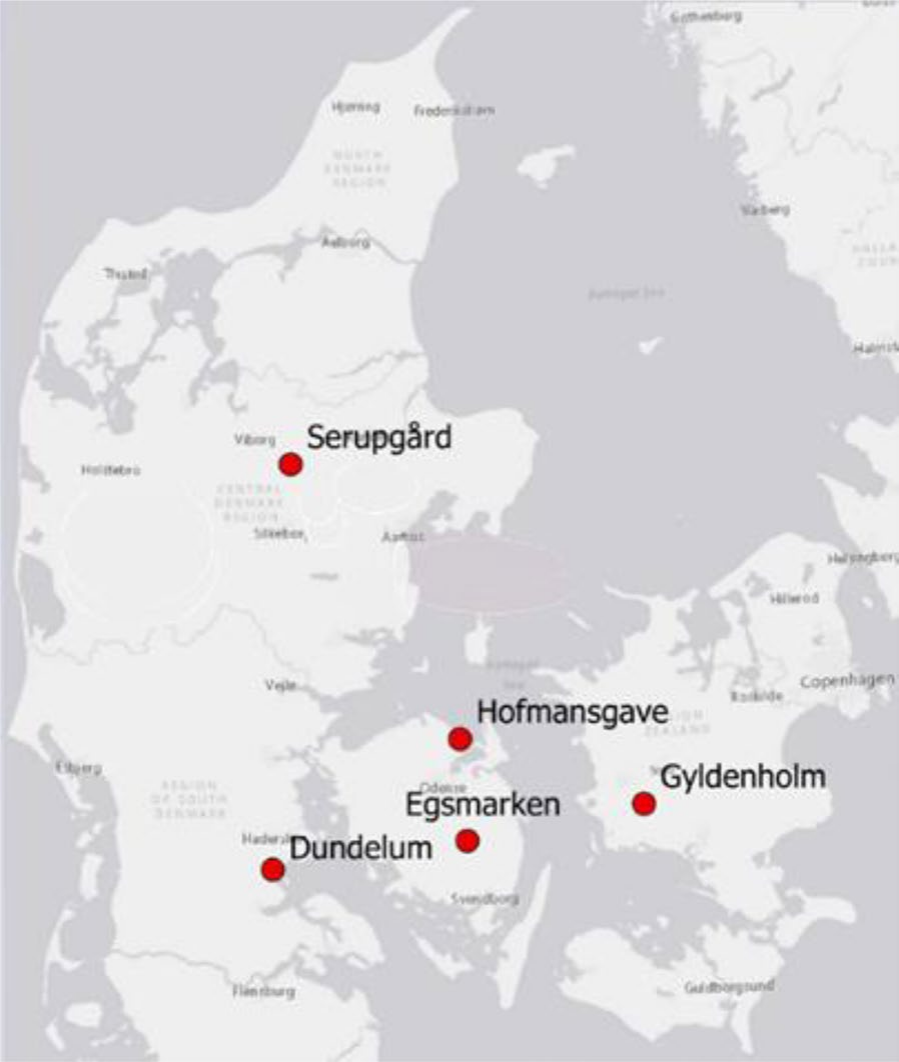
Map showing the location of the five bioreactor facilities

This study includes results from five new bioreactor facilities located in Denmark (Fig. 1). It was a prerequisite from the Agricultural Agency, which funded the project, that the five facilities should be established in different geo-regions in order to assess differences in the final construction design and performance at sites with different yearly precipitation amounts and patterns (Table S1); accordingly, each facility received different hydraulic loads. Further, a requirement for the approval of the construction of the facility was that NO_3_^−^-N concentrations above 4 mg N L^−1^ were measured several times during the discharge season. Four facilities (Gyldenholm, Egsmarken, Dundelum, and Serupgård) were built during the first half of 2018 and came into operation in autumn 2018, while Hofmansgave came into operation in January 2019. The facilities at Gyldenholm and Hofmansgave consisted of three bioreactors (i.e. 3 subunits) running in series, while the other facilities only had one bioreactor. The reason for constructing Gyldenholm and Hofmansgave with three bioreactors running in series was to lead water during periods of low flow to only one reactor to avoid complete reduction of NO_3_^−^ and the reduction of sulphate with the risk of developing nuisance odour as hydrogen sulphide. Hence, with increasing water flow from the drainage catchment drainage, water would successively be led to basin two and three as well. The dimensions and design of each bioreactor are specified in Table 1, and photos of the sites can be found in the Supplementary material (Figs. S1–S5). The bioreactors had a horizontal flow regime except for the Dundelum reactor, which had a vertical (top-down) flow design. The horizontal flow regime was controlled by fixed levels in inlet and outlet wells (Fig. S6) or a dynamic build-up of the hydraulic gradient with an increasing inlet rate because the diameter of the outlet pipe is smaller than the diameter of the inlet pipe (Figs S7–S8, Table 1).

**Table 1:** Characteristics of the bioreactors at the different study sites

Bioreactors		Coordinates		Dimensions	Volume	Flow design^1)^	Total drained catchment	Relative amount of inlet drainage water treated in the bioreactor^2)^	Volume sedimentation pond
		Latitude (°)	Longitude (°)	*W* × *L* × *D* (m)	m^3^		Ha	%	m^3^
Gyldenholm	A	55.331	11.457	16.2 × 21.1 × 1.4	477	Horizontal-a	120	10	4.7
	B	55.3308	11.4571	16.2 × 21.1 × 1.4	477	Horizontal-a		17	
	C	55.3306	11.4571	16.2 × 21.1 × 1.4	477	Horizontal-a		30	
Hofmansgave	A	55.5387	10.4889	22.3 × 15.0 × 1.8	602	Horizontal-a	129	20	111
	B	55.5386	10.4888	22.3 × 15.0 × 1.8	602	Horizontal-a		15	
	C	55.5385	10.4886	22.3 × 15.0 × 1.8	602	Horizontal-a		15	
Egsmarken		55.234	10.5162	13.2 × 33.2 × 1.2	526	Horizontal-b	57	20	135
Dundelum		55.1551	9.4859	13.2 × 41.2 × 1.2	652	Vertical	45–60	33	81
Serupgård		56.3765	9.5984	5.8 × 36.0 × 1.3	271	Horizontal-b	80	13	76

^1)^Horizontal-a. Fixed hydraulic gradient by inlet and outlet well (Fig. S6). Horizontal-b. Hydraulic gradient evolving dynamically (Figs. S7–S8)

^2)^The bioreactor units and subunits did not treat all drainage water from the catchment but only the relative share indicated in this column. For Gyldenholm and Hofmansgave, the share was based on measured amount of inlet water to the reactors, measured total water amount from the catchment and measured bypass flow. For the other three reactors, the relative share was estimated based on drainage maps and drained area compared to the size of the topographic catchment

The volume of the bioreactors varied from 271 to 652 m^3^, reflecting differences in the drainage discharge from the catchment areas. The bioreactors were not dimensioned to receive all the drainage water from the catchment, and at high flow a fraction of the water was therefore bypassed by a manually set damper or pump at the inlet wells to prevent overloading of the bioreactor and thereby aiming at an HRT of minimum 10 h.

All bioreactors except Hofmansgave were equipped with a bentonite geo-membrane (John Hunderup Import & Export, Denmark) to prevent exchange of water between the surrounding soil matrix and the bioreactor. At Hofmansgave, the membrane was made of polypropylene (Junifol, Millag, and Denmark) because the groundwater was saline. All bioreactors were filled with a layer of 1.2–1.8 m filter matrix consisting of 100% willow woodchips (Ny Vraa I/S, DK, chip sizes 0.4–6 cm). This woodchip layer was defined as the active wet filter matrix of the bioreactors. Subsequently, the wet filter matrix layer was topped with an additional unsaturated woodchip layer of 0.3–0.5 m to allow methane oxidation (Carstensen et al., 2019).

A sedimentation pond was located in front of the bioreactors at all the facilities to act as a buffer at peak flow events and trap a fraction of the sediment transported in the tile drainage water towards the bioreactor. All the bioreactors had a re-oxygenation system next to their outlets, consisting of a change in elevation to produce a vertical fall to ensure that the water discharging from the bioreactors was not anoxic when it reached the recipient waters (often low order streams or ditches).

The five facilities were studied during two hydrological years (1 August 2018 to 31 July 2019 and 1 August 2019 to 31 July 2020), later referred to as the hydrological years 2018–2019 and 2019–2020, respectively. Although the mean air temperature in the two hydrological years was relatively similar, varying from 9.1 to 10 °C, the “annual” precipitation differed markedly, being 657–1135 mm in 2018–2019 and 569–859 mm in 2019–2020 (Table S1).

### Monitoring methods

#### Flow and hydraulic residence time

To measure the water flow, the bioreactors were equipped with electromagnetic flowmeters (Waterflux 3070, Krohne, Germany) connected to a data logger (Campbell CR1000, Campbell Sci., Logan, UT, USA) that recorded mean flow velocity every 10 min. A flowmeter was placed at the outlet wells at all bioreactors, but at Hofmansgave and Gyldenholm, a flowmeter was also mounted at an inlet well placed upstream the distribution well, which controlled the amount of bypassing water. In this way, the total bypassed water and N could be calculated.

The hydraulic residence time (HRT) in the present study was calculated as

1 $$HRT={V}_{BR}\times \theta /Q$$

where ${V}_{BR}$ (m^3^) is the water-saturated volume of the bioreactor, $\theta$ (fraction) is the porosity of the bioreactor, and $Q$ is the accumulated discharge of water in the bioreactor over a selected period of time (m^3^/*T*) where *T* is time integrated over the selected period, i.e. the whole period or just a day or an hour with flow. The water level was kept constant as the outlet pipes were fixed at a certain height, and since the hydraulic conductivity in woodchips is very high, only peak flow events might have resulted in a temporary, short increase (hours) of the water level. Porosity was assumed to be 60% based on studies by Bruun et al., ([Bibr CR7][Bibr CR8]).

#### Nutrients, oxygen, and water temperature

We used a mass balance approach to determine the retention or release of solutes in the bioreactors. Flow proportional water samples (100 mL) were taken automatically at all bioreactors by use of a CR1000 datalogger and ISCO water samplers (Teledyne ISCO, Inc., NE, USA) installed at the inlet and outlet. At Hofmansgave and Gyldenholm, the datalogger triggered the ISCO samplers to sample when the accumulated water flow reached 720 m^3^ of water at the main inlet and for every 240 m^3^ of water from each of the outlets from the three subunits. The ISCO samplers kept the sampled water refrigerated at 4 °C. At Egsmarken, Dundelum and Serupgård, the datalogger triggered the ISCO sampler (ISCO 6712 Full-size Portable Sampler, Teledyne ISCO, Inc., NE, USA) to take a sample for every 120 m^3^ of flow from the bioreactor. Samples were collected every second week and transported in an icebox to the laboratory. Upon arrival, the water samples were kept at 4 °C until analysis for TN and total phosphorus (TP). All TN samples were analysed using a Technicon AutoAnalyzer according to DS 221 (1975) and TP by the photometric method according to DS 292 (1985). In this study, nutrient removal is expressed as the quantity of nutrients removed per m^3^ of the filter matrix per year (g N m^−3^ yr^−1^) and per day (g N m^−3^ d^−1^). The latter was obtained when normalised with the number of days with flow (Table 2).

**Table 2:** Hydraulic residence time (HRT), water temperature, and N and P concentrations (inlet) at the bioreactors

Bioreactors	Monitoring period	HRT^3)^	Days with flow	Drainage water temperature	Inlet TN concentration	Inlet TP concentration
		Mean [min] (h)		Yearly mean [min–max] (°C)	Yearly mean [min–max] (mg L^–1^)	Yearly mean [min–max] (mg L^–1^)
Gyldenholm A	2018–2019^1)^	10 [3]	36	6.3 [4.6–7.9]	20.6 [8.9–26.9]	
	2019–2020	28 [3]	138	7.9 [6.2–12.3]	14.6 [12.7–17.1]	0.057 [0.025–0.21]
Gyldenholm B	2018–2019^1)^	13 [3]	30	6.5 [4.9–8]	20.2 [8.9–26.9]	
	2019–2020	13 [2]	121	8.2 [6.5–12.7]	14.9 [12.7–17.1]	0.048 [0.025–0.21]
Gyldenholm C	2018–2019^1)^	26 [7]	138	7.2 [4.9–11.8]	21.6 [8.9–26.9]	
	2019–2020	20 [6]	191	8.2 [6.5–12.3]	13.8 [12.7–17.1]	0.055 [0.025–0.21]
Hofmansgave A	2018–2019^2)^	50 [6]	69	7.8 [3.8–13.7]	11.2 [6–16.1]	0.045 [0.025–0.18]
	2019–2020^2)^	30 [8]	63	7.7 [4.5–13.7]	11.5 [7.6–12.7]	0.064 [0.04–0.11]
Hofmansgave B	2018–2019^2)^	53 [5]	46	8.7 [3.9–15.6]	11.3 [6–16.1]	0.046 [0.025–0.18]
	2019–2020^2)^	43 [6]	48	7.8 [4.5–13.7]	12.2 [7.6–12.7]	0.067 [0.04–0.11]
Hofmansgave C	2018–2019^2)^	69 [5]	49	7.8 [4–13.2]	10.3 [6–16.1]	0.045 [0.025–0.18]
	2019–2020^2)^	31 [6]	56	7.8 [4.5–13.7]	11.3 [7.6–12.7]	0.030 [0.04–0.11]
Egsmarken	2018–2019	64 [12]	107	6.4 [4.3–9.9]	12.2 [8.3–24.6]	0.070 [0.029–0.15]
	2019–2020	59 [4]	217	7.7 [5.5–12.7]	13.8 [9.9–20.7]	0.099 [0.026–0.31]
Dundelum	2018–2019	70 [4]	129	6.9 [4.6–11.2]	8.9 [7.1–13.4]	0.268 [0.065–1.4]
	2019–2020	29 [7]	202	6.4 [2.4–11.6]	10.0 [5.3–16.7]	0.245 [0.098–0.82]
Serupgård	2018–2019	63 [5]	129	6.1 [4.8–8.5]	28.2 [13.7–46.4]	0.269 [0.19–1.4]
	2019–2020	32 [5]	188	6.2 [4–10.8]	14.5 [5.6–21.5]	0.156 [0.083–0.39]
Mean		39 [5]	109	7.3 [4.7–11.9]	14.1 [8.6–20.1]	0.112 [0.045–0.347]

^1)^Sampling of TP failed

^2)^The Hofmansgave facility was only in operation from February to April in both monitoring periods due to technical problems

^3)^Mean HRT was calculated using Eq. 1 for the whole monitoring period with flow. The number of days with flow and the accumulated flow for the hydraulic year are given in Tables 2 and 3, respectively, while minimum HRT was based on the daily HRT

### Cost analysis

The purpose of the economic analyses was to estimate the required investment and the yearly costs of a bioreactor. Based on this and the effect of the bioreactors, the cost efficiency with respect to mainly N removal could be calculated and compared to other reduction measures (Eriksen et al., 2020; Jacobsen & Gachango, 2013).

The economic calculations were based on the actual investments and expected running costs involved in the project; the costs are given in Danish Krone (DKK) and US Dollar ($), where DKK 100 = $14.41 (exchange rate on 25.4.2022). The estimated lifetime of the investment was 20 years, and the discount rate used was 4% following the socioeconomic guidelines from the Danish Ministry of Finance (Finansministeriet, 2019). This gives an annuity factor of 0.0736, which is the annual cost of an investment of 1 unit for 20 years with an interest rate of 4%. The data on investments, based on actual agreements made with sub-contractors, and running costs were provided by Aarhus University (F. Plauborg, personal communication). The investment costs covered the construction of the facility, including, for instance, soil removal and pipe laying. In some cases, additional wells were established (see Table S4 in the Supplementary material). The running costs include electricity, loss of income, and costs of cleaning the pipes.

The lost income concerned the area taken out of production, here often around 0.3–0.4% of the catchment area, including a sedimentation pond and a free area for moving the machinery used to maintain the bioreactors, e.g. in connection with addition of supplementary woodchips over time. The area taken out for free water surface-constructed wetlands was around 1%. The loss of income was set to $271 per ha, following the national average (Eriksen et al., 2020). Should the project be stopped after 20 years, money is needed to re-establish the area, but this was not included in the calculations. Also, if the project is continued after 20 years, pond redesign may be required.

The average height of the biomaterial (woodchips) used in our cost analysis was 1.7 m due to the requirement of an unsaturated layer of 0.3–0.5 m woodchips on the top to promote methane oxidation. With time, the volume of the biomaterial will shrink, requiring addition of another 0.5 m layer of biomaterial roughly every 6 years (C.C. Hoffmann, personal communication). The average price of the biomaterial used was DKK 134 ($19) t^−1^, which is 8% cheaper than the price level used in the N catalogue of DKK 145 ($21) t^−1^ (Eriksen et al., 2020).

The running costs included additional biomaterial (woodchips), supposing addition of 0.5 m woodchips on top of the bioreactor every sixth year to compensate for loss of material (following the approach adopted in Eriksen et al., 2020). The adding of extra woodchips every sixth year is based on observations (C.C. Hoffmann, personal communication) that the woodchip level is lowered around 0.1 m yearly due degradation processes. For some locations, the running costs of the bioreactor also included flushing the inlet pipes every second year for sediments and algae blooms. In low-lying areas, a pump may be needed, involving electricity costs.

The advisory part includes a description of the idea, inspection of the site, and elaborating a draft project layout. It also comprises elaboration of a detailed project plan, contract agreements with contractors and project follow-up until the final delivery, including coordination between contractors, but the costs stated by the contractors do not include permission application to authorities. In our case, catchment advisors were in charge of finding locations and obtaining permissions. For other projects, these costs could be paid by the system of special advisors to help promote constructed wetlands. In our case, the service provided by the catchment officers was paid by the research project, but it is omitted from the other calculations as it is free to farmers. It is difficult to establish whether Aarhus University had more need for private consultants than most farmers, but it is not unlikely as these sites were public projects.

In our analyses, costs and cost efficiency were compared to the standard costs found in the guidelines from the Agricultural Agency regarding support for new bioreactors (Agricultural Agency, 2020). These standard costs are based on 30 project estimates for 11 bioreactor locations (Agricultural Agency, 2019), resulting in the amounts shown in the Supplementary material, Table S2, and they are used to estimate the investment support that should be given to a constructed wetland project.

## Results and discussion

### Performance of the bioreactors during the hydrological years 2018–2020

#### Hydraulic residence time, water temperature, and nutrient concentrations

The different bioreactors have different volumes and treat different relative amounts of inflowing tile drainage water from the catchments (Table 1). As a result, the mean HRT—calculated over the flowing season—varied markedly between the bioreactors with a mean HRT of 10 h at bioreactor Gyldenholm A to 70 h at Dundelum (Table 2). The bioreactors were designed to enable control of the inlet rate, aiming at a minimum HRT of 10 h. For the reactors at Serupgård, Dundelum, Egsmarken, and Hofmansgave, control involved manual change of a shutter and at Gyldenholm modification of the pumping rate. However, this control was not fully successful as evidenced by the daily HRT with values as low as 2 h (Table 2). At very low inflow rates, the yearly max HRT increased to unrealistically high values, and these are therefore omitted from Table 2. It should be noted that the number of days with inflow of tile drainage water to the bioreactors differed greatly between the five sites, from 30 to 217 days (Table 2). For all bioreactors, the average yearly mean inlet TN concentration ranged between 9.6 and 24.7 mg N L^−1^, while the yearly mean TP varied from 0.035 to 0.54 P mg L^−1^ (Table 2).

Basins C and A at Gyldenholm and Hofmansgave, respectively, were the first basin in a series of three to receive water at low flow, and the number of days with flow was therefore higher for these basins than for the two other basins at Gyldenholm and Hofmansgave. This was obtained by use of a smaller inlet pipe diameter and a lower inlet level than for the two other basins (Plauborg et al., 2021). The idea was, as described above, that low water flow from the drainage catchments was directed to only one basin and not to all basins to reduce the risk of complete reduction of NO_3_^−^, reduction of sulphate (Carstensen et al., 2019), and a possible release of hydrogen sulphide.

### Total nitrogen

In this and the following section, we present a synthesis of nutrient removal results. All the bioreactors removed TN during the study period, the average removal being 30.5% of the TN load (Table 3). TN removal rates in the treated water (i.e. not bypassed) varied largely amongst the bioreactors (6–55%) and between the two hydrological years (Table 3). However, the hydraulic load of the bioreactors also showed large variations (17–229 m^3^ m^−3^ bioreactor yr^−1^), and it should be noted that the hydrological year 2018–2019 was relatively dry, which also impacted the TN load at the bioreactor inlets, except for Gyldenholm.

**Table 3:** Water flow, total nitrogen (TN) load, and TN removal efficiency and rates per cubic metre active bioreactor

Bioreactors	Hydrological year	Water flow	Yearly TN load inlet	TN removal efficiency	Yearly TN removal rate	Daily TN load inlet^1)^	Daily TN removal rate^2)^
		m^3^ m^−3^ yr^−1^	g N m^−3^ yr^−1^	%	g N m^−3^ yr^−1^	g N m^−3^ d^−1^	g N m^−3^ d^−1^
Gyldenholm A	2018–2019	86	1776	5.5	97.00	49.33	2.69
	2019–2020	117	1706	23.8	406.00	12.36	2.94
Gyldenholm B	2018–2019	55	1111	8.8	97.50	37.03	3.25
	2019–2020	229	3405	19.1	649.50	28.14	5.37
Gyldenholm C	2018–2019	139	3012	20.5	617.00	21.83	4.47
	2019–2020	229	3161	22.2	700.20	16.55	3.67
Hofmansgave A	2018–2019^3)^	33	368	30.2	111.00	5.33	1.61
	2019–2020^3)^	50	579	26.6	154.00	9.19	2.44
Hofmansgave B	2018–2019^3)^	21	238	29.8	71.00	5.17	1.54
	2019–2020^3)^	27	329	41.9	138.00	6.85	2.88
Hofmansgave C	2018–2019^3)^	17	175	54.6	95.50	3.57	1.95
	2019–2020^3)^	44	496	38.8	192.50	8.86	3.44
Egsmarken	2018–2019	40	487	43.9	214.00	3.78	1.66
	2019–2020	89	1224	32.9	402.50	6.06	1.99
Dundelum	2018–2019	44	392	49.0	192.00	3.04	1.49
	2019–2020	170	1702	46.0	782.50	9.05	4.16
Serupgård	2018–2019	49	1383	31.4	434.00	12.93	4.06
	2019–2020	140	2025	23.4	473.50	9.33	2.18
Mean	All years	88	1325	30.5	327.33	13.88	2.90

^1)^Calculated as the yearly load rate divided by the number of days with water flow, cf. Table 2

^2)^Calculated as the yearly removal rate divided by the number of days with water flow, cf. Table 2

^3)^The Hofmansgave facility was only in operation from February to April in both monitoring periods due to technical problems

**Fig. 2:**
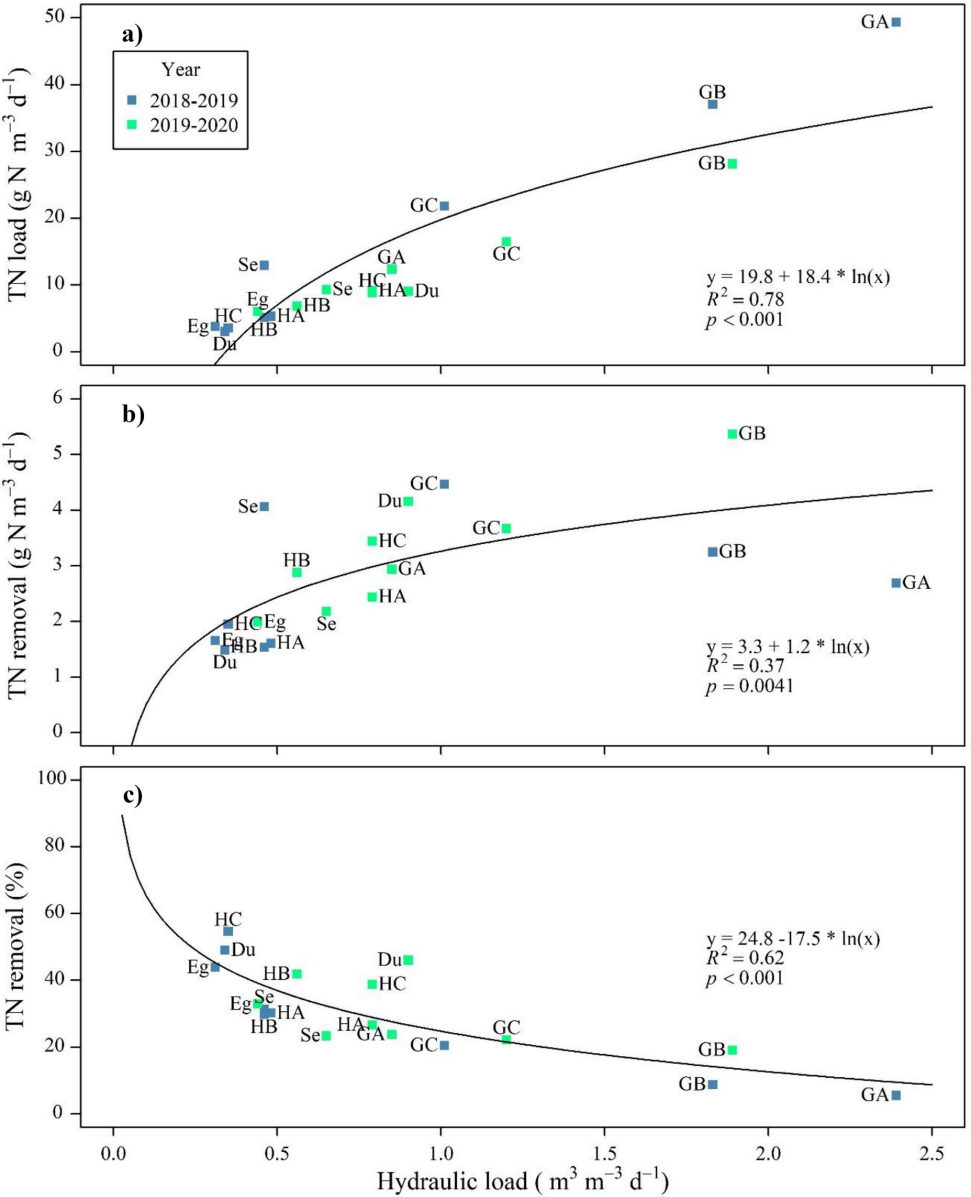
Top: total nitrogen (TN) load per day at the inlet of the bioreactors relative to hydraulic load per day. Middle: TN removal (total removal per day) relative to hydraulic load per day. Bottom: TN removal (percent removal per day) relative to hydraulic load per day. The relationships between the variables were assessed using linear regressions before and after transformation of the hydraulic load using natural logarithms. Legends GA, GB, and GC are Gyldenholm A, B, and C, respectively. HA, HB, and HC are Hofmansgave A, B, and C, respectively. Se is Serupgård. Eg is Egsmarken. Du is Dundelum

We hypothesised that the dry period had lowered the removal rates in 2018–2019 and, as expected, there was a clear increase in the TN load with an increasing hydraulic load to the bioreactors (Fig. 2, top). At the same time, there was an increase in TN removal rates (in g N m^−3^ d^−1^) to the bioreactors at increasing hydraulic load (and thus TN load) (Fig. 2, middle). However, a negative relationship was observed between TN load and TN removal efficiency (as percent TN removal) (Fig. 2, bottom). Hence, higher TN removal (in g N m^−3^ d^−1^) was achieved at lower efficiency (percent TN). Thus, both the percent efficiency and the absolute TN removal should be considered when evaluating the annual, seasonal, and daily performance of different bioreactors.

The reported bioreactors received only agricultural drainage water in the 4–6 autumn, winter and spring months, and even at low flow situations, in this period, we did not sense any smell of hydrogen sulphide, but as described by Hoffmann et al. (2019) and Carstensen et al. (2019), water flow in summertime with higher temperatures could result in 100% removal of N and hence reduction of sulphate to hydrogen sulphide.

Even though the HRT was as low as 2 h in some periods, the N removal in the field-based bioreactors equalled that recorded for woodchip bioreactors in Denmark, Canada, USA, and New Zealand (Table 4), located in similar climatic conditions. The composition of the filter matrix differed between the studies (Table 4), but all the included matrices were based on wood products (sawdust or woodchips) with or without addition of gravel, sand, or soil. Overall, the comparison of our results with international studies suggests that the design of the Danish bioreactors provides a satisfactory efficiency of NO_3_^−^ removal from agricultural drainage water. In a recent review of the performance of 27 bioreactors, Christianson et al. (2021) reported higher average N removal rates with a median of 5.1 g N m^−3^ d^−1^ and a mean ± SD of 7.2 ± 9.6 g N m^−3^ d^−1^. However, not all of these 27 bioreactors treated agricultural drainage water, e.g. some with an N removal rate between 4 and 8 g N m^−3^ d^−1^ treated water from aquaculture. For other bioreactors with an N removal rate between 6 and 8 g N m^−3^ d^−1^, HRT could not be reported.

**Table 4:** Nitrogen removal rates in field-based woodchip bioreactors treating agricultural drainage water

Reference	Country	Composition filter matrix	Nitrogen removal rate g N m^−3^ d^−1^	Monitoring period
This project, all bioreactors	Denmark	See the “Materials and methods” section	1.49–5.37^1)^	2 years
Hoffmann et al. (2019), horizontal flow	Denmark	Woodchips + mussel shells 50:50	2.03–2.10	2 years
Hoffmann et al. (2019), horizontal flow	Denmark	Woodchips + mussel shells 75:25	2.20–2.22	2 years
Hoffmann et al. (2019), vertical upwards flow	Denmark	Woodchips + mussel shells 50:50	1.67–2.14	2 years
Hoffmann et al. (2019), vertical upwards flow	Denmark	Woodchips + mussel shells 75:25	1.77–2.15	2 years
Hoffmann et al. (2019), vertical downwards flow	Denmark	Woodchips + mussel shells 50:50	2.16–2.21	2 years
Hoffmann et al. (2019), vertical downwards flow	Denmark	Woodchips + mussel shells 75:25	1.16–1.79	2 years
Christianson et al. (2012)	USA	Woodchips (60%) + gravel	0.38–3.78	7 years
Christianson et al. (2012)	USA	Woodchips (100%)	0.86–1.56	2 years
Christianson et al. (2012)	USA	Mixed hardwood chips	0.41–7.76	3 years
Christianson et al. (2012)	USA	Woodchips (100%)	0.42–5.02	2 years
Elgood et al. (2010) ^2)^	Canada	Pine woodchips (100%)	0.69	1.1 years
Jaynes et al. (2008)	USA	Oak woodchips	0.62	5 years
Schipper et al. (2005)	New Zealand	Pine sawdust (30%) + soil	1.4	9 days
Schipper et al. (2010)	New Zealand	Coarse sawdust + woodchips 50:50	1.4	1 year
Schipper et al. (2010)		Sawdust + woodchips 50:50	5–10	2 years
Schipper et al. (2010)	New Zealand	Sawdust + woodchips 50:50	0–11	1.4 year
Schmidt and Clark (2012)	USA	Pine sawdust (50%) + quartz sand	4.9–5.5	1.8 years
van Driel et al. (2006)^2)^	USA	Fine and coarse wood particles	2.66	2.2 years
van Driel et al. (2006)^2)^	USA	Fine and coarse wood particles	0.76	1.7 years
Woli et al. (2010)	USA	Woodchips mixed	6.4	2–3 years

^1)^Only TN was measured, but in general NO_3_^−^-N constitutes about 90–95% of TN in Danish drainage water (Blicher-Mathiesen et al., 2015)

^2)^Transformed to volumetric units

The first row is total nitrogen (TN), all other results show nitrate (NO_3_^−^-N) removal

### Total phosphorus

TP retention was generally negative as TP was lost from the different bioreactors, but for some reactors, the retention was positive in the second year of operation (Table 5).

**Table 5:** Total phosphorus load and retention by the bioreactors

Bioreactors	Hydrological year	Yearly TP load inlet	TP retention efficiency	Yearly TP retention rate	Daily TP load inlet	Daily TP retention rate
		g P m^−3^ yr^−1^	%	g P m^−3^ yr^−1^	mg P m^−3^ d^−1^	mg P m^−3^ d^−1^
Gyldenholm A	2018–2019^1)^					
	2019–2020	6.7	− 13	− 0.87	48.6	− 6.3
Gyldenholm B	2018–2019^1)^					
	2019–2020	11	− 0.4	− 0.04	90.9	− 0.4
Gyldenholm C	2018–2019^1)^					
	2019–2020	13.7	52	7.12	71.7	37.3
Hofmansgave A	2018–2019^2)^	1.50	− 4097.9	− 61.47	21.7	− 890.8
	2019–2020^2)^	3.20	− 1730.9	− 55.39	50.8	− 879.2
Hofmansgave B	2018–2019^2)^	0.96	− 3549.8	− 34.08	20.9	− 740.8
	2019–2020^2)^	1.80	− 2233.3	− 40.20	37.5	− 837.5
Hofmansgave C	2018–2019^2)^	0.77	− 4992.6	− 38.44	15.7	− 784.6
	2019–2020^2)^	1.30	− 2626.0	− 34.14	23.2	− 609.6
Egsmarken	2018–2019	2.80	− 1374.8	− 38.49	21.7	− 298.4
	2019–2020	8.80	27.9	2.46	43.6	12.2
Dundelum	2018–2019	11.80	− 844.4	− 99.64	91.5	− 772.4
	2019–2020	41.70	34.8	14.51	221.8	77.2
Serupgård	2018–2019	13.20	− 240.2	− 31.71	123.4	− 296.3
	2019–2020	21.80	43.4	9.46	100.5	43.6
Mean	All years	9.40	− 1436.4	− 26.73	65.6	− 396.4

^1)^Sampling of TP failed

^2)^The Hofmansgave facility was only in operation from February to April in both monitoring periods due to technical problems. In the beginning of the second year, all woodchips were removed and stored, and a new membrane was installed. Hereafter, woodchips were filled into the bioreactor again

**Fig. 3:**
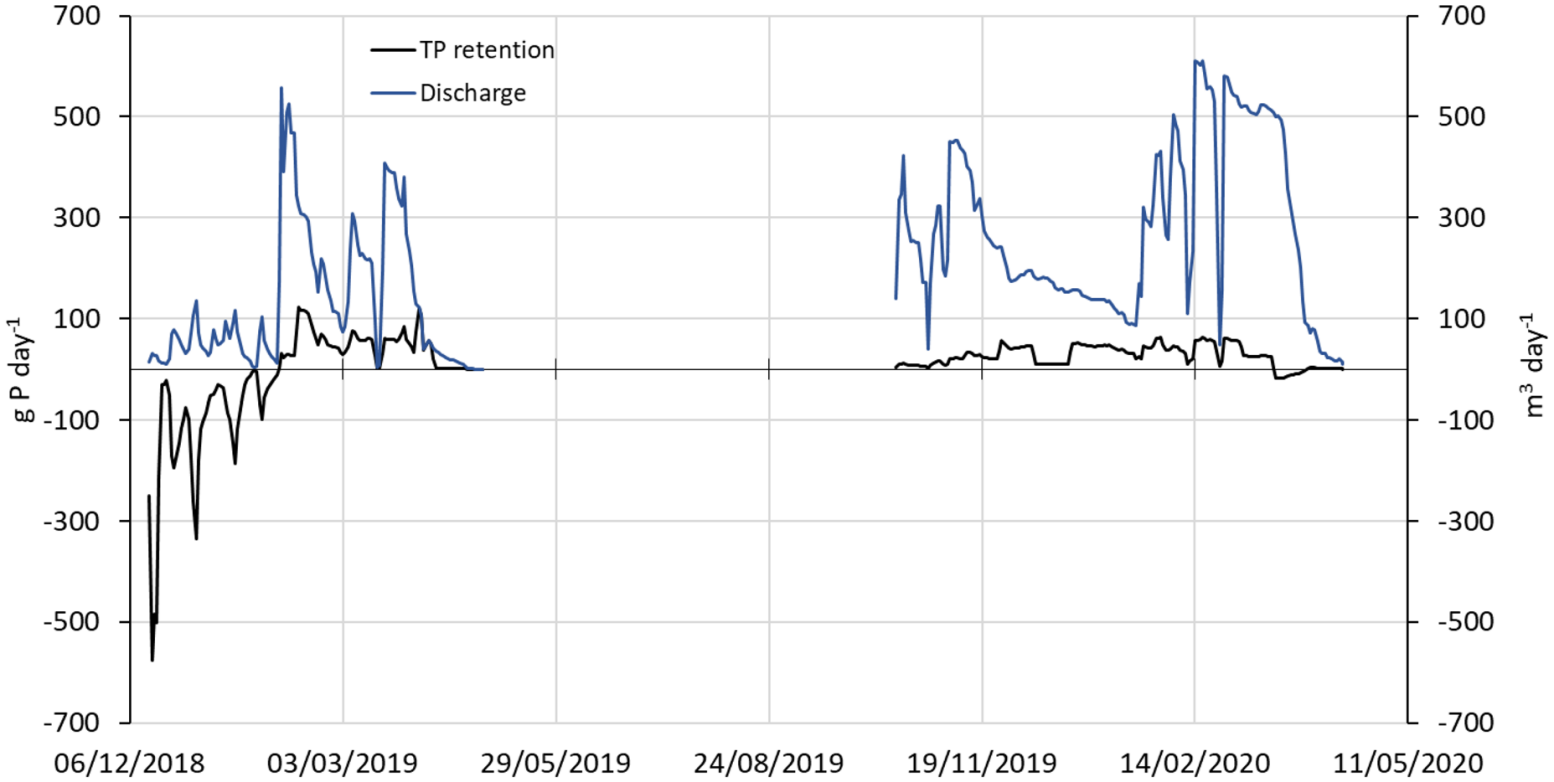
Total P release (black line) and hydraulic load (blue line) from the bioreactor at Serupgård during the two hydrological years 2018–2019 and 2019–2020

At Serupgård, Egsmarken, and Dundelum, the negative TP retention (or release) was especially noticeable in the first months following bioreactor establishment, e.g. as shown for Serupgård (Fig. 3). Overall, the bioreactors monitored in the project had a mean TP retention of − 26.73 g P m^−3^ yr^−1^ or − 396.4 mg P m^−3^ d^−1^, indicating P loss to the recipients.

Overall, TP retention by the bioreactors varied between − 31.71 and − 99.64 g P m^−3^ year^−1^ in their first year of operation. This is in line with the results of Carstensen et al. (2019), who in a 5-year study of bioreactors also found loss of TP in the first year, which could be attributed to significant PO_4_^3−^-P losses, while particulate P was retained during the whole 5-year monitoring period. The bioreactors in the present study were established in spring 2018, a dry year with almost absence of precipitation between May and November. Leaving a bioreactor with fresh and moist woodchips under unsaturated conditions in a dry summer and an autumn probably promoted composting of the woodchips and sped up the mineralisation process. As displayed in Fig. 3, the release of TP decreased to zero over a relatively short period (2–3 months), whereas release of PO_4_^3−^-P lasted up to nine months in the study by Carstensen et al. (2019). At Hofmansgave, TP release was observed in both years, probably due to the technical problems and the handling of the woodchips. Thus, at the start of the second year, all woodchips had to be removed and placed on the soil surface under aerobic conditions when a new membrane was installed, after which the woodchips were backfilled. Overall, the TP release seemed limited in time, and several bioreactors turned from being a P source during the first year to acting as a P sink in the remaining monitoring period (Table 4), which is in agreement with Carstensen et al. (2019). However, the TP release in the first year was still substantial and could potentially impact vulnerable downstream recipients. It is therefore important to take into consideration the risk of P release for downstream waterbodies, especially when implementing several bioreactors in the same catchment. To mitigate this P release, use of technical solutions such as the addition of material to trap P (e.g. sand filter) may be a possibility. Further, in temperate regions, bioreactors should be established in autumn or winter, the optimal timing being immediately before the start of the drainage season. Hence, most of the woodchips will be water saturated, and immediately afterwards anaerobic processes will take over.

### Cost efficiency analysis

In the economic analyses, Gyldenholm was treated as one bioreactor, and Hofmansgave was left out as the data did not cover two hydrological years due to shift of the membrane (see Table S3 basic costs data). The standard costs mentioned above also form the basis for the cost efficiency calculations in the Danish overview of nitrogen measures (the N catalogue), which compares a range of possible measures to reduce nitrogen losses to the sea (Eriksen et al., 2020). As shown in Table S3, the relative size of the reactor was around 0.3% of the effective catchment area, which is a bit larger than in the N catalogue where the fraction is set to 0.2% of the catchment area (Eriksen et al., 2020).

The size of the bioreactor relative to the catchment area is still quite low compared to that of constructed open wetlands where the wetland area constitutes around 1% of the catchment area. The results on bioreactor efficiency presented here are based on only two years of data from the four bioreactors. To consolidate the results and hence the economic calculations, several more years of monitoring are required.

The investments are described in more detail in Table S4, the costs related to the establishment of the work site being the first. The establishment costs are often independent of the size of the reactor, whereas the other investments often increase with reactor size. In our calculations, additional investments were included to cover extra wells (flushing wells), biomaterial and costs related to advisory tasks. It should be noted that costs related to permissions and archaeological diggings were not included; they are frequently an issue and therefore included in the standard costs. The consultancy fee is around 20% of the costs, but as no two sites are the same, the need for advice differs from site to site.

At Gyldenholm, it was not considered necessary to install a pump, but it appeared that a pump was in fact necessary as the main inlet drain pipe was buried surprisingly deep into the ground. The installation of a variable frequency pump in a 7-m deep well cost DKK 496,000 ($71,474), resulting in a yearly cost of DKK 36,500 ($5,260), which can be perceived as an additional, but perhaps not required, cost.

Table 6 shows the calculated annual costs of which the largest item is related to the initial investment in bioreactor construction and biomaterial, while the second largest item is the advisory costs.

**Table 6:** Annual costs of a bioreactor (DKK yr^−1^)

Name of reactor	Serupgård	Egsmarken	Dundelum	Gyldenholm	Share of total (%)
Bioreactor and initial biomaterial^1**)**^	26,548	45,855	47,883	112,067	57
Additional well^1**)**^	756	1041	0	0	0
Additional pump^1**)**^	0	0	0	36,504	9
Additional biomaterial^1**)**^	4458	9420	12,582	20,125	11
Electricity etc	0	0	0	20,000	5
Cleaning pipes	1500	1500	1500	1000	1
Loss of income	102	198	246	539	0
Consultant^1**)**^	11,916	10,531	10,531	29,727	15
Total costs	45,280	68,546	72,742	219,962	100
Total costs without pump	45,280	68,546	72,742	163,458	86

For Gyldenholm, the running costs related to pump electricity were DKK 20,000 yr^−1^ and moving of the lawn DKK 1000. For Serupgård, Egsmarken, and Dundelum, the running costs were DKK 1,500 y^−1^ for drain cleaning. The annual costs of the investments marked with ^1)^ are based on 20 years and 4% interest (see also Table S4). DKK 100 = $14.41 (exchange rate on 25.4.2022)

The additional purchase of more biomaterial comes next. If a pump is needed, the costs increase as is the case for Gyldenholm where pump investment and electricity increase the costs, potentially also the consultancy costs.

Table S5 shows the result of scaling the annual costs in Table 6 to a reactor size of 0.2 ha. This conversion ensures that the costs across reactors are based on the same size. The figures in Table S5 show that the cost efficiency for the four reactors varies between DKK 230 and 342 per kg N ($33 to 49 per kg N) The cost efficiency figures presented in Table 7 show some but not large differences in the N reduction (standard effect for constructed wetlands (CW) of 0.2 ha) between the sites when they are scaled to the same size. It is, however, worth noticing that the N effect at three of the four sites differs between the two years. Thus, the effect in year 2 is much higher at these three sites, which is partly due to the weather, but the removal percentages in year 1 and year 2 are fairly similar. Some of the basins at Gyldenholm have a low removal percentage, reducing the average. In the present study, the costs per kg N removed varied from DKK 273 to 423 (Table 7) ($40–61 per kg N).

**Table 7:** N and P reduction and cost efficiency for woodchip bioreactors

Name of reactor	Serupgård	Egsmarken	Dundelum	Gyldenholm
Reactor size (m^2^)	271	526	652	1431
TN reduction (kg N/year)	123	162	318	612
Standard effect (kg N/yr/0.2 CW ha)	908	617	975	855
TN removal %	27	38	48	17
TP reduction 1^st^ year (kg P/yr)	-9	-20	-70	-30
TP reduction 2^nd^ year (kg P/yr)	3	1	9	3
Cost efficiency (DKK/kg N)	368	423	273	359
Cost efficiency (DKK/kg N) without pump	368	423	273	300
Cost efficiency (DKK/kg P)	17.415	52.727	8.266	73.321

The P effect is typically negative for the first year since P from the biomaterial is released into the nearby stream, so the year 2 effect was used instead. TN removal and efficiency are based on Table 3. TN effect is based on the average effect listed in Table 3 multiplied by the volume of Serupgård (434 + 473.5 g N m^−3^ yr^−1^)/2* 271 m^3^ * 1000 = 123 kg N yr^−1^. Note: DKK 100 = $14.41 (exchange rate on 25.4.2022)

The P effect is negative in the first year and subsequently positive (see Table 7). It will, therefore, often take several years before the amount removed will equal the amount of P lost during year 1. The P reduction is limited to around 1–9 kg P per year, and the P removal ranges between 21 and 43%. The P cost efficiency calculations range from DKK 8300 to 7300 per kg P ($1,196 to 1052 per kg P) based on year 2.

In a study by Christianson et al. (2021), investments of $5,000 to $27,000 (DKK 35,000–190,000) were reported, with an estimated cost efficiency varying between $2.50 and $20 per kg N (DKK 17 to 138 per kg N) for a range of different projects mainly in the US. This is under half the costs calculated in this study. Other findings from New Zealand indicate costs around $6–7 per kg N (DKK 42–49 per kg N) using only a 10-year life span of the reactor, which increases the annual costs of the investment (Sarris & Burbery, 2018). For a bioreactor in Australia, White et al. (2022) estimated expenses of $14 (DKK 95) per kg NO_3_-N removed, which is around 22 to 32% of the costs calculated in our study. This is partly due to a much lower investment in the Australian study (DKK 35,000 compared to around DKK 500,000) than that in the Danish bioreactors (around 600 m^2^ in size).

Moreover, costs related to replacement of biomaterial and advisory costs were not included in the analysis by White et al. (2022). However, further studies are needed to compare the applied factors in the cost effectiveness analyses to consolidate the big differences. It seems that the key difference is that the Danish investment is much higher than in other cases around the world. It is not clear if additional biomaterial is included in the US cases. This accounts for 10% of the investment in the Danish cases (see Table S4). It is noted in Christianson et al. (2021) that advisory costs vary from around $640 (4,500 DKK) for two days and eight hours to $7500 (52,000 DKK) for reactors designed by private design firms. Our study shows that the Danish advisory costs were 2–3 times higher than even those of private design firms in the USA.

We compared the project costs to the standard costs reported by Danish authorities (see Table S5), including the costs of woodchip bioreactors given in the Danish guidelines for the smallest (500 m^2^) and largest site (2500 m^2^) (Eriksen et al., 2020). These guideline costs were included due to the low number of existing woodchip bioreactors, implying that the guideline costs could help to improve standard figures as well as to identify specifically where the costs of the analysed sites were larger than expected. In the comparison, the bioreactors were all adjusted to the same size (0.2 ha).

In general, the investments were larger than expected even when using the smallest case of 500 m^2^ in the N catalogue (Eriksen et al., 2020). They were also much larger than anticipated in previous analyses (Jacobsen & Gachango, 2013). This suggests that the investments may be higher than expected although the investments based on the standard costs given in Table S2 are also derived from real projects. In some cases, additional wells and pumps can generate additional costs, which may significantly increase the costs per year. It seems that the advisory costs in the four projects were much larger than the standard costs. This could perhaps be due to higher complexity and limited assistance from the catchment officers as ours was also a research project. Catchment officers emphasise that the administrative costs included in the standard costs in Table S2 only include the most needed tasks and thus not all project coordination issues.

**Fig. 4:**
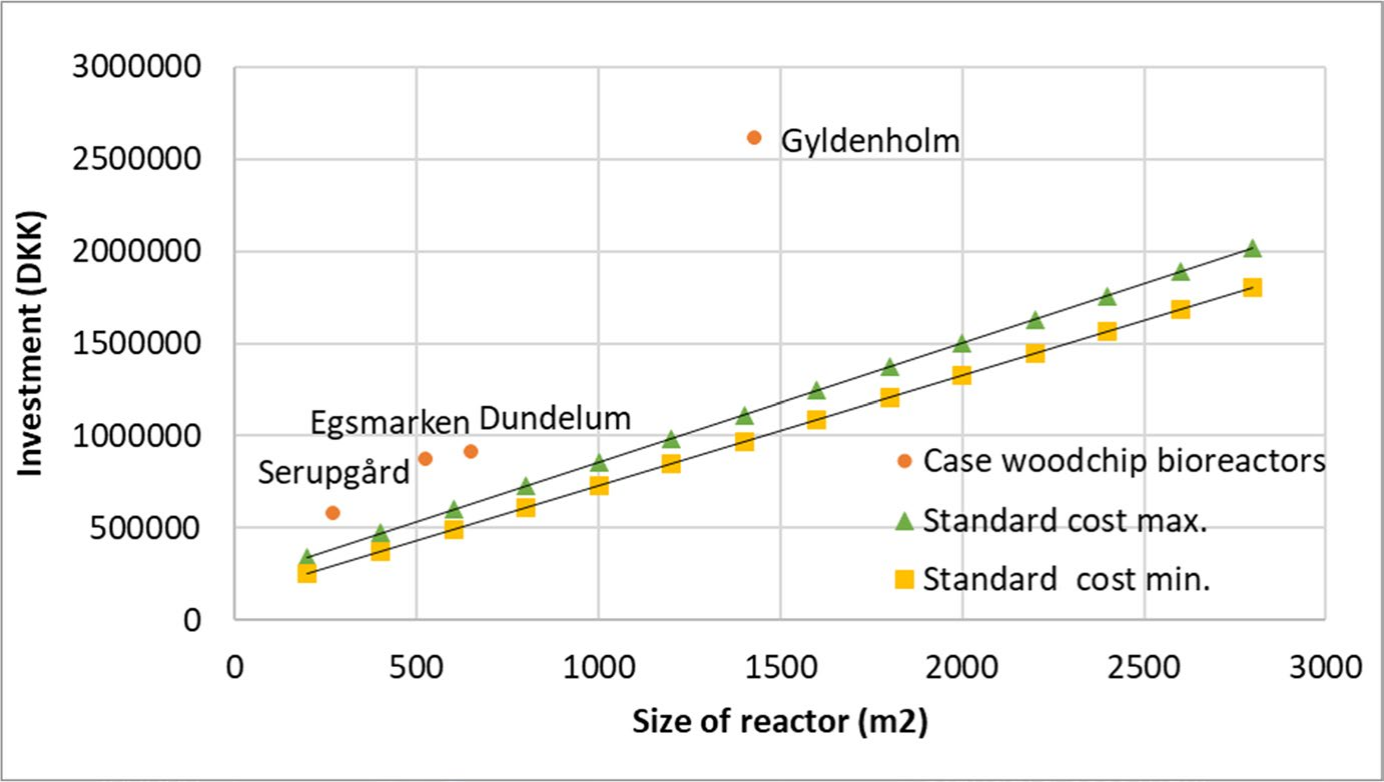
Investments in woodchip bioreactors depending on reactor size. Investments in biomaterial over time are included for all projects. Minimum standard costs exclude investment in pumps, but this is included in the maximum standard costs, standard cost min

In Fig. 4, investments in the project bioreactors were compared with the basic investments in the Danish Agricultural Agency guideline (Table S5). The investments required were the lowest for the smaller reactors, but small reactor investments were the highest in the same-size comparisons (0.2 ha).

The larger investments in the projects analysed in this paper could be related to the adopted pilot approach and the fact that the companies involved had not engaged in this type of project before. Our project shows that no two sites are the same; thus, identification and management of the best location may take time. Furthermore, the costs in the guide from the Agricultural Agency may be somewhat conservative to avoid overcompensation.

The initial bioreactor is supplemented with 0.5 m biomaterial in years 6, 12, and 18. Catchment officers suggested that the initial level of biomaterial should rather be 2.25 m (and not 1.7 m) to follow the requirements set by the Agricultural Agency. The advisory costs are much larger than expected, possibly related to the pilot nature of the project. Aarhus University had almost the same help from catchment officers that farmers might have received. Normally, farmers undertake project coordination themselves, but in our project, this was carried out by a consultant at a cost that the farmer might not be compensated for. This indicates that, in many projects, the consultancy costs may be higher than the amount included in the standard costs. This is also because the farmer does not want to bear the responsibility for the final acceptance of the project by the Agricultural Agency.

The costs of comparable measures such as traditional wetlands are 34–39 DKK ($5–6) per kg N, and the costs of other agricultural measures like catch crops are 7–167 DKK ($1–24) per kg N and for set-a-side 24–96 DKK ($3–14) per kg N measured in the root zone. In comparisons with the effect of bioreactors, groundwater retention must be included. With a retention of 70%, this would increase the costs of e.g. catch crops to 23–556 DKK ($3–80) per kg N. When using a mix of cost efficient measures, the average costs of meeting the Danish WFD targets were estimated to approx. 80 DKK ($12) per kg N (Jacobsen, 2022). So, the costs of the bioreactors are clearly higher than the average costs of measures in Denmark.

## Conclusions

The performance of five field-based bioreactor facilities, two bioreactors with three subunits, and three with a single bioreactor, was reported for two hydrological years. The TN removal rate varied from 1.49 to 5.37 g N m^−3^ d^−1^, with an overall average of 2.90 g N m^−3^ d^−1^. The yearly N removal efficiency varied from 6 to 55%, with a mean of 30.5% across bioreactors and years. In the first year of operation, the bioreactors had a TP loss rate of 298.4 to 890.8 mg P m^−3^ d^−1^. However, in the second year, most bioreactors retained phosphorous at rates varying from 12.2 to 77.2 mg P m^−3^ d^−1^.

Overall, the annual costs of the four bioreactors in the present project were about twice the average costs compared to other nitrogen reduction measures (Eriksen et al., 2020).

The higher costs of the bioreactors were mainly due to larger investments and higher consultancy costs and, in some cases, also investment in an additional pump. The reduction of costs per kg N (with pump) based on the actual size was, on average, ca. DKK 350 ($50) per kg N, which is around 50% higher than the standard costs used by the Danish authorities for bioreactor establishment. The cost efficiency linked to the P removal in year 2 showed large variations from around DKK 8000 to 73,000 ($1153 to 10,519) per kg P for the four bioreactor facilities.

## Supplementary Information

Below is the link to the electronic supplementary material.

Supplementary file1 (DOCX 4366 KB)

## Data Availability

The datasets generated during and/or analysed during the current study are available from the corresponding author upon reasonable request.
