# Supplementary material for: Cost effectiveness, nitrogen, and phosphorus removal in field-based woodchip bioreactors treating agricultural drainage water
Source: Environ Monit Assess. 2023 Jun 16;195(7):849. doi: 10.1007/s10661-023-11358-8 (PMC10275798; doi:10.1007/s10661-023-11358-8)
Supplement: Supplementary file 1 — Supplementary file1 (DOCX 4366 KB) [file 10661_2023_11358_MOESM1_ESM.docx]

**Title:**

Cost effectiveness, nitrogen and phosphorus removal in field-based woodchip bioreactors treating agricultural drainage water

**SUPPLEMENTARY MATERIALS**

**Table S1** Air temperature and precipitation during the hydrological years.

| Facility | Hydrological year | Air Temperature | Precipitation |
| --- | --- | --- | --- |
|  |  | mean (°C) | sum (mm) |
| Gyldenholm | 2018-2019 | 9.9 | 569 |
|  | 2019-2020 | 10 | 657 |
| Hofmansgave | 2018-2019 | 9.7 | 544 |
|  | 2019-2020 | 9.7 | 812 |
| Egsmarken | 2018-2019 | 9.8 | 617 |
|  | 2019-2020 | 9.7 | 893 |
| Dundelum | 2018-2019 | 9.7 | 706 |
|  | 2019-2020 | 9.7 | 1135 |
| Serupgård | 2018-2019 | 9.2 | 809 |
|  | 2019-2020 | 9.1 | 1083 |

**Table S2** Standard costs used by the Danish Agricultural Agency.

| Item | Base price (DKK) | Additional costs DKK pr. m^2^ |
| --- | --- | --- |
| Obligatory parts (incl. bio material). | 105,000 | 350 |
| Pump | 69,000 | 45 |
| Path | 8,000 | - |
| Advisory costs | 13,250 | - |
| Permissions | 6,200 | - |
| Archaeological examinations | 11,800 | 2.5 |
| Additional biomaterial | - | DKK 145 m^3^ |

Source: Agricultural Agency (2020)

**Table S3** Basic data for the four bioreactors analysed.

| Name of reactor | Serupgård | Egsmarken | Dundelum | Gyldenholm | Average |
| --- | --- | --- | --- | --- | --- |
| Size of reactor (m^3^) | 271 | 526 | 652 | 1431 | 720 |
| Complete catchment area (ha) | 80 | 57 | 53 | 120 | 78 |
| Size of reactor compared to complete area (%) | 0.03 | 0.09 | 0.12 | 0.12 | 0.09 |
| Effective catchment area (ha)^1)^ | 10.4 | 11.4 | 17.49 | 68.4 | 26.9 |
| Effective catchment, a (%) of the complete catchment^2)^ | 13 | 20 | 33 | 57 | 34 |
| Size of reactor compared to effective area (%) | 0.26 | 0.46 | 0.37 | 0.21 | 0.33 |
| Area taken out of production (reactor x2) (ha) | 0.04 | 0.1 | 0.1 | 0.3 | 0.1 |
| Height of biomaterial at the beginning (m) | 1.7 | 1.7 | 1.7 | 1.7 | 1.7 |
| Height of additional biomaterial (m) | 0.5 | 0.5 | 0.5 | 0.5 | 0.5 |
| Biomaterial initial (m^3^) | 466 | 889 | 1,140 | 2,485 | 1,245 |
| Biomaterial (DKK/m^3^) | 130 | 144 | 150 | 110 | 134 |
| Cost of area taken out of production (DKK/ha) | 1,883 | 1,883 | 1,883 | 1,883 | 1,883 |

Source: Pers. comm. Finn Plauborg, 2020, and own calculations. For Dundelum, an average was used (45+60 ha)/2. **^1)^** The effective catchment area is a percent of the complete catchment area. **^2)^** The percent effective catchment is equal to the relative amount of drainage water treated, cf. Table 1.

**Table S4** The base investment in the bioreactors (DKK)

| Name of reactor | Serup-gård | Egsmarken | Dundelum | Gyldenholm | Share of total cost |
| --- | --- | --- | --- | --- | --- |
| Establishing the work area | 52,500 | 95,000 | 100,000 | 326,260 | 12% |
| Moving soil | 110,676 | 260,597 | 196,994 | 358,055 | 19% |
| Electricity | 52,044 | 69,575 | 90,250 | 156,215 | 7% |
| Wells | 85,000 | 70,000 | 92,500 | 409,000 | 13% |
| Base investment | 300,219 | 495,172 | 479,744 | 1,249,530 | 51% |
| Biomaterial initial | 60,580 | 128,016 | 171,000 | 273,500 | 13% |
| Additional well | 10,271 | 14,152 | 0 | 0 | 10% |
| Additional pump | 0 | 0 | 0 | 496,107 | 4% |
| Biomaterial (yr. 6, 12 and 18) | 42,050 | 90,407 | 116,733 | 187,883 | 9% |
| Consultant | 161,947 | 143,123 | 143,123 | 404,000 | 17% |
| Other^1)^ | 0 | 0 | 0 | 0 | 0% |
| Total | 575,067 | 870,870 | 910,600 | 2,611,020 | 100% |

Note : **^1)^** No additional costs for identification of sites were included as this is assumed to be a task carried out by the catchment officers. Paths, archaeological digs and permissions are not encumbered with costs.

**Table S5** Comparison of key figures between bioreactors and the N catalogue**^1)^** based on a standard size reactor of 2,000 m^2^ (0.2 ha).

|  | N catalogue  500 m^2^ | N catalogue  2500 m^2^ | Serupgård | Egsmarken | Dundelum | Gyldenholm |
| --- | --- | --- | --- | --- | --- | --- |
| Size of reactor at outset (m^2^) | 500 | 2500 | 209 | 526 | 652 | 1431 |
| Scaling factor | 4,0 | 0,8 | 7,4 | 3,8 | 3,1 | 1,4 |
| Annual costs: |  |  |  |  |  |  |
| Bioreactor and initial biomaterial | 59,233 | 59,233 | 146,635 | 152,704 | 130,906 | 153,556 |
| Additional well | 0 | 0 | 5,578 | 3,959 | 0 | 0 |
| Additional pump | 26,931 | 10,684 | 0 | 0 | 0 | 51,019 |
| Additional biomaterial | 36,276 | 36,276 | 32,897 | 35,816 | 38,597 | 28,127 |
| Archaeological digs | 1,236 | 1,236 | 0 | 0 | 0 | 0 |
| Electricity etc. | 0 | 0 | 0 | 0 | 0 | 27,952 |
| Establishing a path | 589 | 589 | 0 | 0 | 0 | 0 |
| Cleaning pipes | 0 | 0 | 11,070 | 5,703 | 1,500 | 1,000 |
| Loss of income | 753 | 753 | 753 | 753 | 753 | 753 |
| -Archaeological digs | 1,236 | 1,236 | 0 | 0 | 0 | 0 |
| Path and permissions | 1,045 | 1,045 | 0 | 0 | 0 | 0 |
| Consultant | 975 | 975 | 11,916 | 10,531 | 10,531 | 29,727 |
| Total costs | 128,274 | 112,027 | 208,849 | 209,467 | 182,287 | 292,135 |
| Total costs without pump | 101,343 | 101,343 | 208,849 | 209,467 | 182,287 | 241,115 |
| Cost efficiency: |  |  |  |  |  |  |
| N reduction (kg N/yr) | 600 | 750 | 908 | 617 | 975 | 855 |
| Cost efficiency (DKK/kg N) | 214 | 149 | 230 | 340 | 187 | 342 |
| Cost efficiency (DKK/kg N) without pump | 169 | 135 | 230 | 340 | 187 | 282 |

The costs are scaled to 0.2 ha (see scaling factor).

Additional economic support: For area in rotation: DKK 35,000 per ha biofilter or DKK 7,000 per 0.2 ha and maintenance DKK 24,000 0.2 ha for 10 years.

**^1)^** The nitrogen catalogue is an overview on potential measures to reduce nitrogen losses to the sea.

Source: Eriksen et al. (2020).

In the calculations in Table S5 all costs – except those related to consultants, paths and permissions – were scaled according to size (0.2 ha). To further improve the scaling, an initial investment of DKK 105,000 was assumed irrespective of size, based on the initial investments shown in Table S4. The additional investments were then scaled according to size. The costs related to archaeological digs were scaled following the agricultural agency guideline, involving a tendency of cost overestimation for smaller bioreactors when these were converted to a size of 0.2 ha.

## **Photos of the sites**


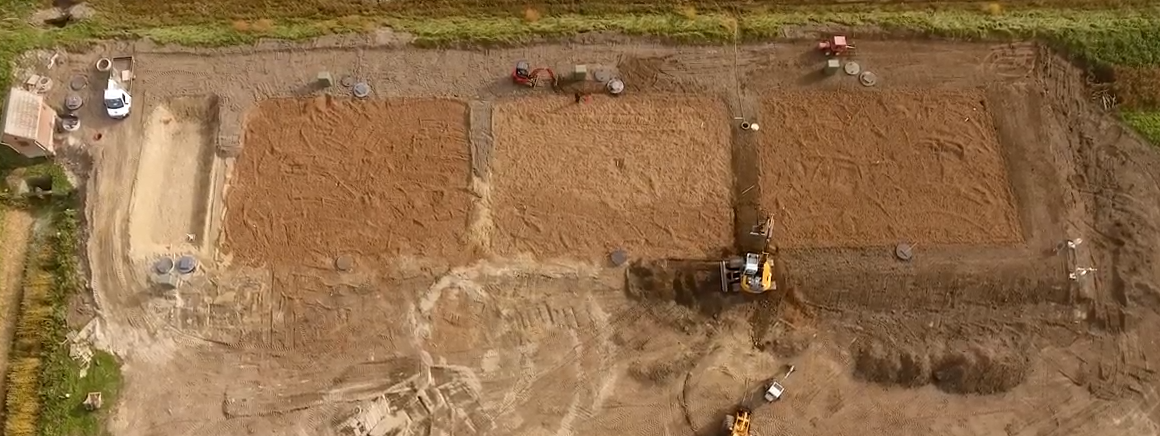


**Fig. S1** The bioreactor facility at Hofmansgave. The sedimentation pond at the left and the three bioreactors A. B and C from left to right can be seen. Picture taken shortly before opening of the drains. Credit: SEGES.


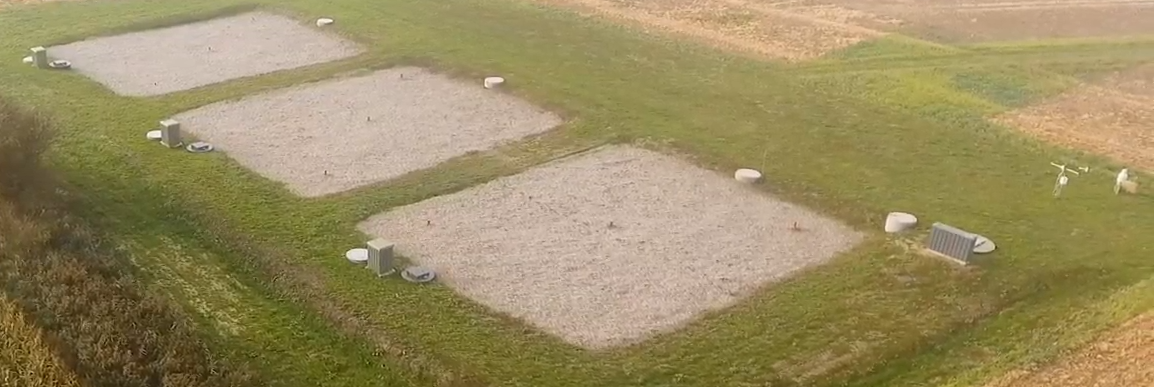


**Fig. S2** The bioreactor facility at Gyldenholm. The three bioreactors A. B and C (left to right) can be seen. Credit: SEGES.


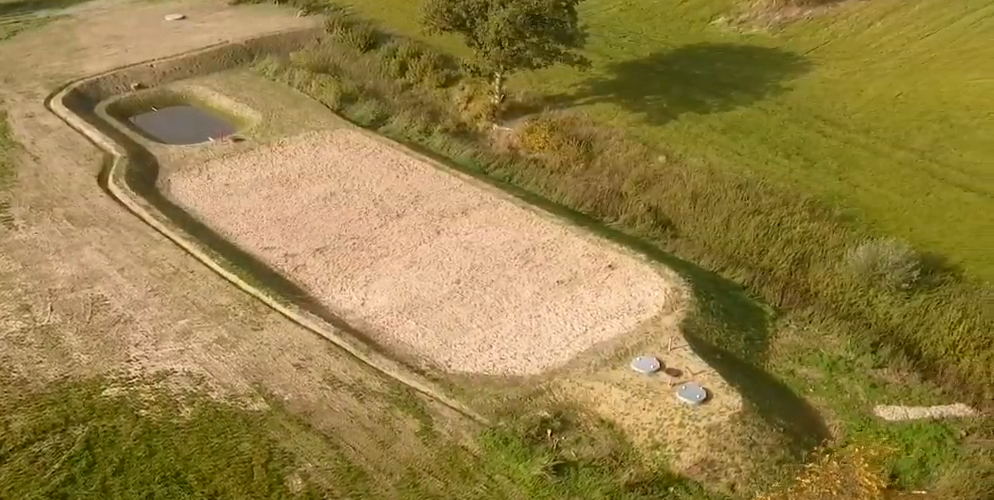


**Fig. S3** The bioreactor facility at Egsmarken. Credit: SEGES.


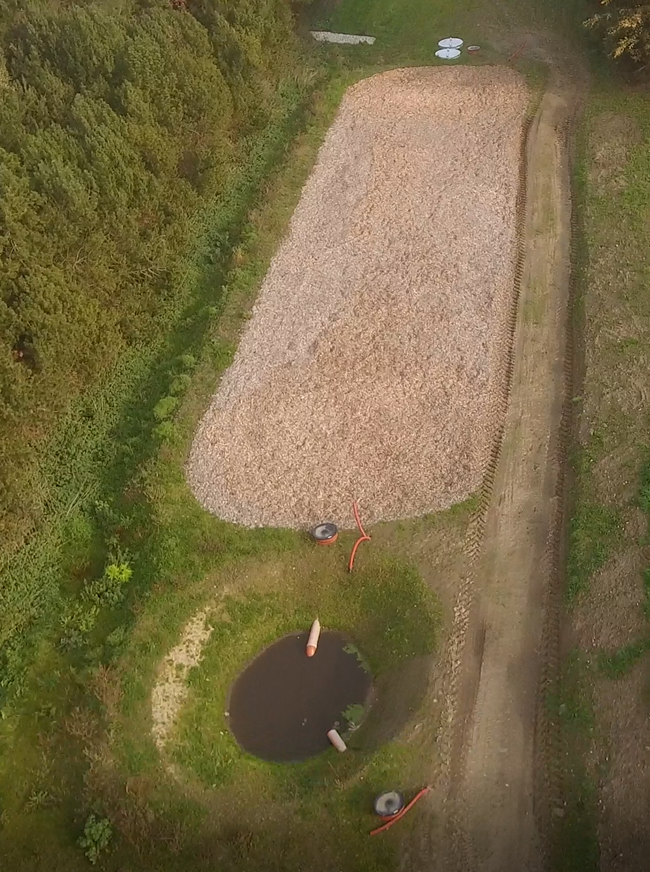


**Fig. S4** The bioreactor facility at Serupgård. Credit: SEGES.


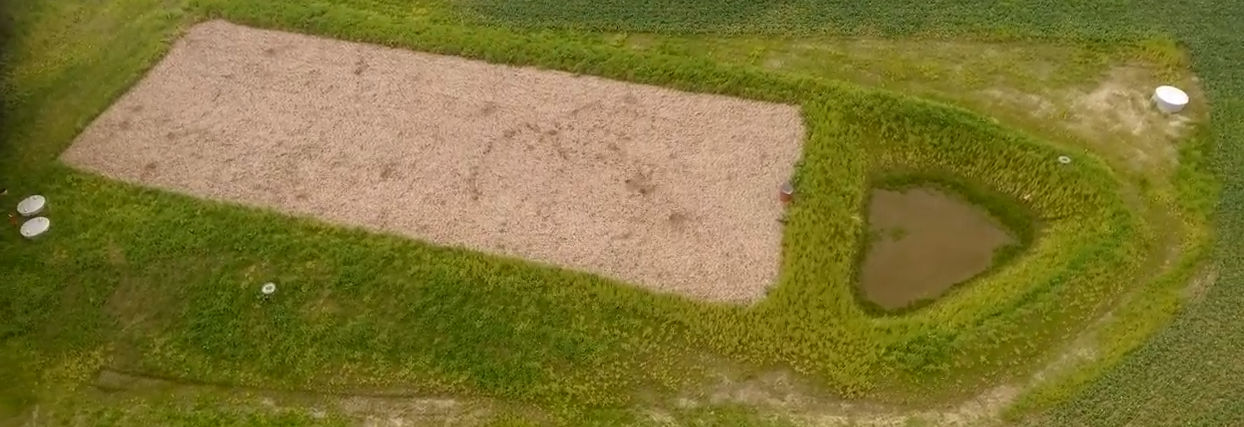


**Fig. S5** The bioreactor facility at Dundelum. Credit: SEGES.


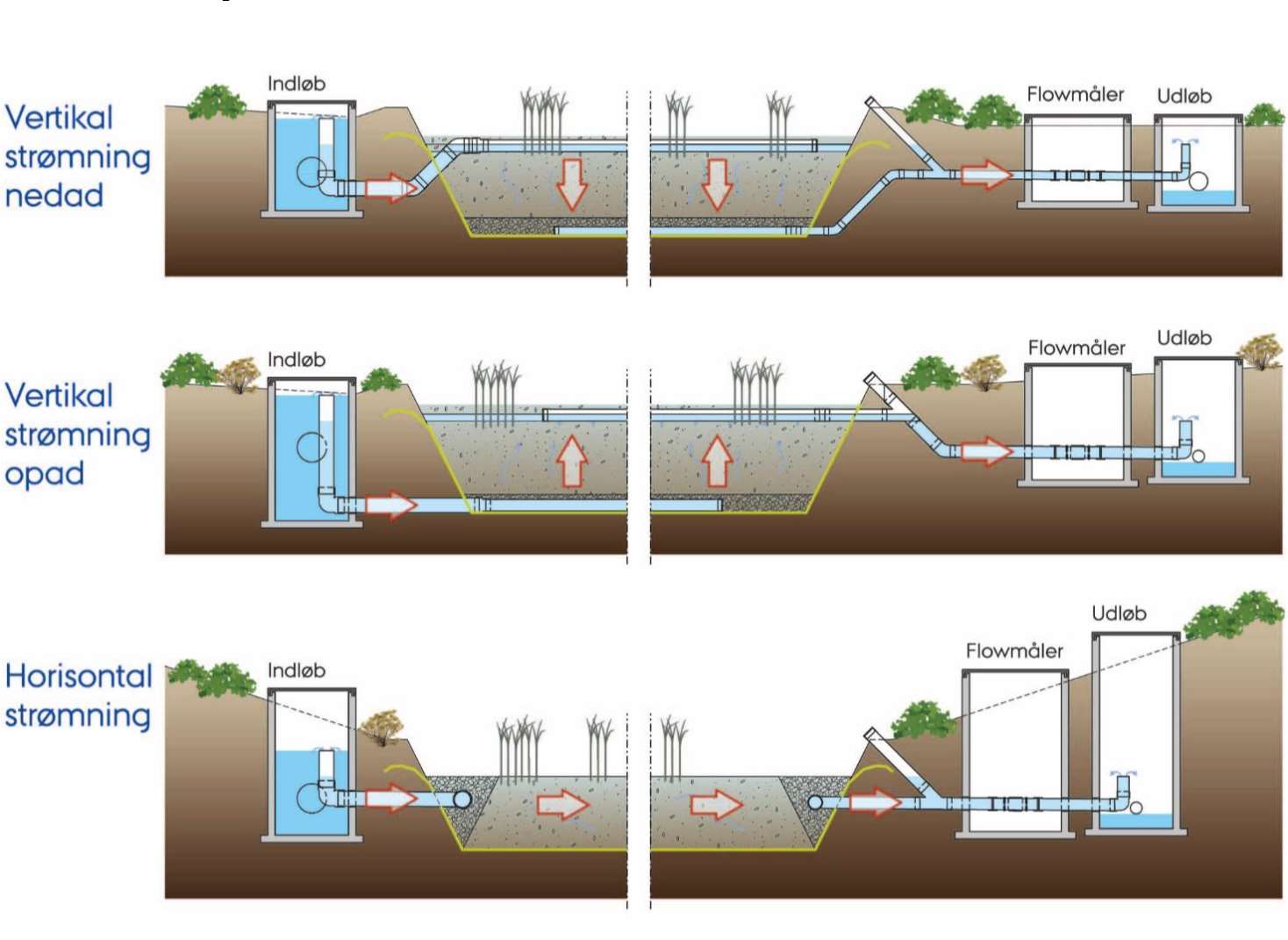


Inlet

Flow unit

**Fig. S6.** Woodchip bioreactor with a horizontal flow regime with inlet and outlet (Hoffmann et al., 2019).


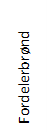

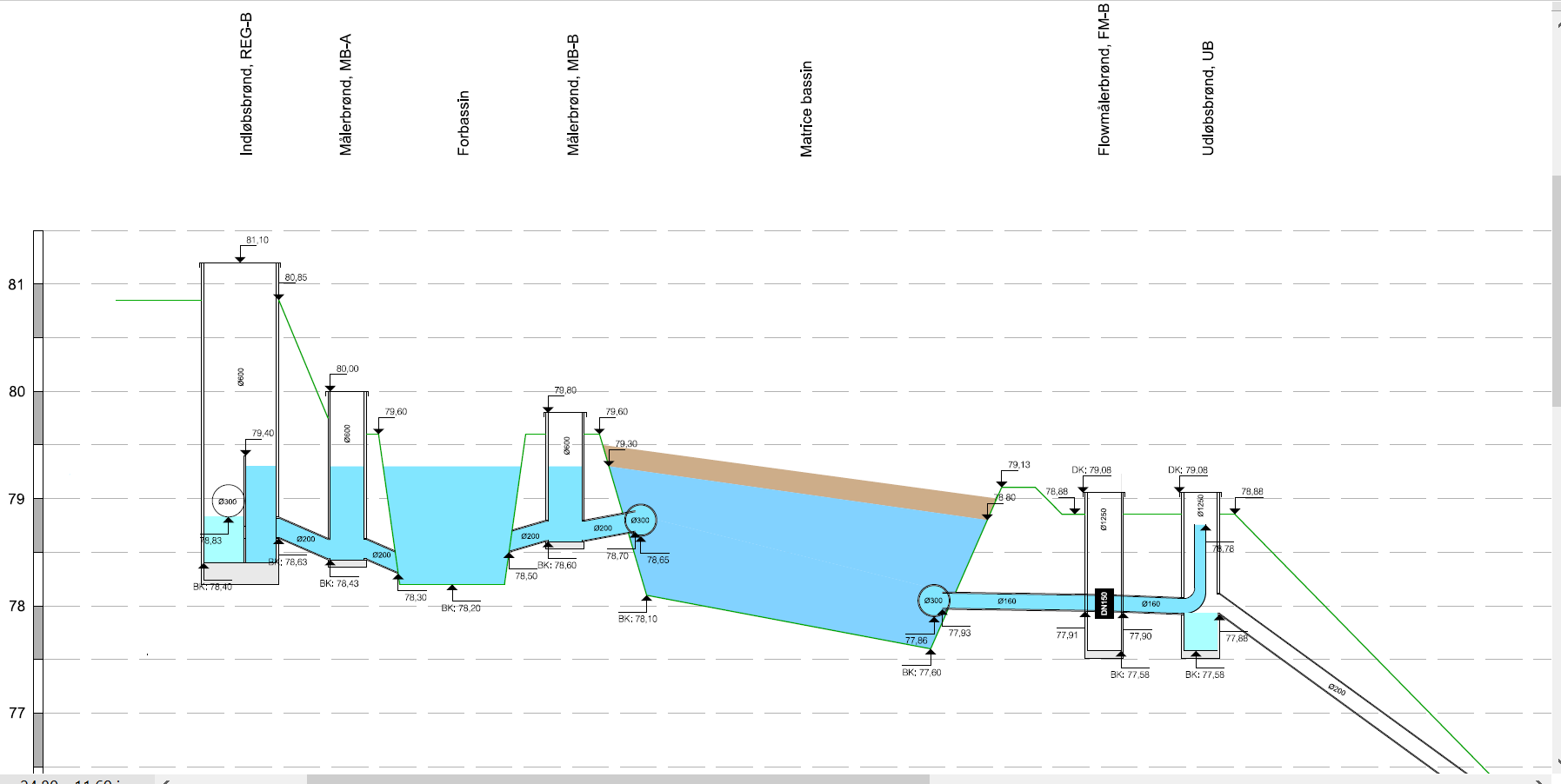


B

A

C

**Fig. S7** Technical drawing of the hydraulics for the plant at Egsmarken on Funen. The red arrows A, B and C mark wells established for sampling and research. These do not have to be established in practice (source: WSP).

B

C

A

**--**


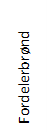

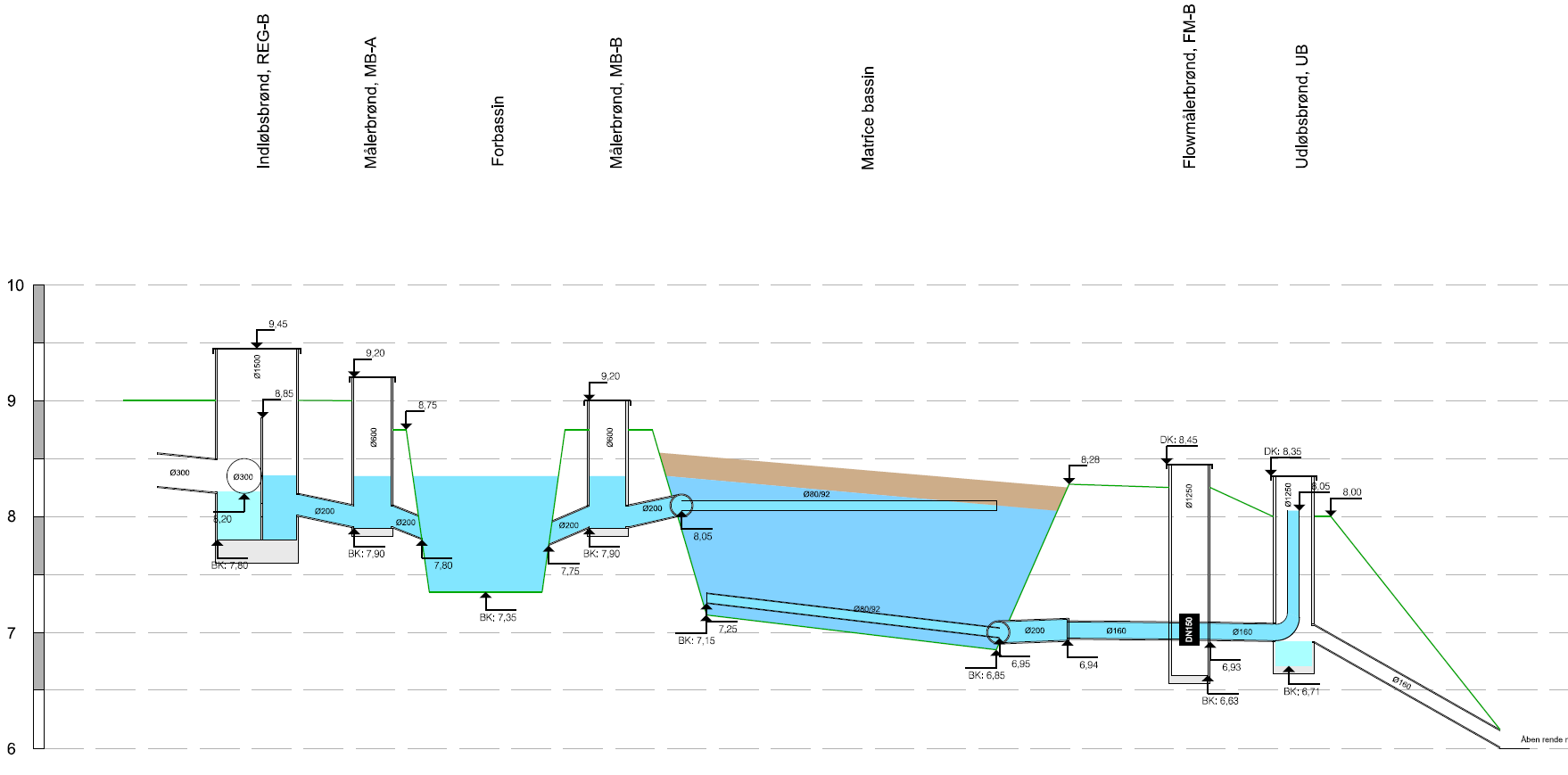


C

B

A

**Fig. S8** Technical drawing of the hydraulics for the plant at Dundelum near Haderslev. The red arrows A, B and C mark wells established for sampling and research. These do not have to be established in practice. The level of the three drainage pipes at the top and bottom in the entire longitudinal direction of the basin is shown (source: WSP).
